# Recent Advances in Regulating Ceramic Monolithic Catalyst Structure for Preferential Oxidation of CO in H_2_

**DOI:** 10.3390/molecules29153481

**Published:** 2024-07-25

**Authors:** Qing Wang, Jiancai Sui, Linlin Li, Yongxiao Tuo, Wenfa Zhang, Guoyu Zhong, Huanxin Zhou, Xiang Feng

**Affiliations:** 1 College of Chemistry and Chemical Engineering, China University of Petroleum (East China), Qingdao 266580, China; s22030083@s.upc.edu.cn (Q.W.); 19854219916@163.com (L.L.); 19854219925@163.com (H.Z.); xiangfeng@upc.edu.cn (X.F.); 2 National Engineering Research Center of Coal Gasification and Coal-Based Advanced Materials, Shandong Energy Group Co., Ltd., Jinan 250101, China; 3 College of New Energy, China University of Petroleum (East China), Qingdao 266580, China; zwf2517039323@outlook.com; 4 Key Laboratory of Distributed Energy Systems of Guangdong Province, Dongguan University of Technology, Dongguan 523808, China; zhonggy@dgut.edu.cn

**Keywords:** hydrogen, CO preferential oxidation, ceramic monolith, Pt-based catalyst, structure regulation

## Abstract

Preferential oxidation of CO (CO-PROX) has tremendous significance in purifying hydrogen for fuel cells to avoid catalyst poisoning by CO molecules. Traditional powder catalysts face numerous challenges, including high pressure drop, aggregation tendency, hotspot formation, poor mass and heat transfer efficiency, and inadequate thermal stability. Accordingly, ceramic monolithic catalysts, known as their excellent thermal stability, high surface area, and superior mass and heat transfer characteristics, are gaining increasing research attention. This review examines recent studies on ceramic monolithic catalysts in CO-PROX, placing emphasis on the regulation of active sites (e.g., precious metals like Pt and Au, and non-precious metals like CuO and CeO_2_), monolith structures, and coating strategies. In addition, the structure–catalytic performance relationships, as well as the potential and limitations of different ceramic monolithic catalysts in practical application, are discussed. Finally, the challenges of monolithic catalysts and future research prospects in CO-PROX reactions are highlighted.

## 1. Introduction

With the growing global demand for clean energy, proton exchange membrane fuel cells (PEMFCs) have gained significant attention due to their high energy conversion efficiency and environmental friendliness. Given the potent toxicity of CO to the anode catalyst of PEMFCs, the effective removal of trace amounts of CO from the hydrogen source of PEMFCs is of paramount importance [1,2]. CO preferential oxidation (CO-PROX) is considered to be one of the most cost-effective and efficient methods for reducing CO concentrations to the ppm level [3], selectively oxidizing CO by introducing a small amount of oxygen or air into hydrogen-rich gas. However, hotspot phenomena occurring on particulate catalysts under high temperature conditions may trigger the reverse water–gas shift reaction, resulting in an increased CO concentration at the outlet [4,5]. The development of ceramic monolithic catalysts with excellent mass transfer and heat transfer properties is particularly important, as they can effectively inhibit the formation of hotspots and enhance reaction efficiency. Moreover, the superior anti-aggregation properties, low pressure drop, and high mechanical strength of ceramic monolithic catalysts facilitate their adaptation to actual industrial conditions.

The ceramic monolithic catalyst primarily consists of a support and a washcoating. The support is typically made from materials such as cordierite or alumina, known for their high-temperature stability, high mechanical strength, and chemical inertness. This support structure features multiple parallel channels or network channels. The typical diameter and height of ceramic monolithic catalysts range from 10 to 20 mm. These monoliths feature a pore density of 400 cells per square inch (cpsi), a wall thickness of 0.15 mm, and a channel width of 1.12 mm. Additionally, the washcoating thickness is less than 0.1 mm. The unique three-dimensional structure of ceramic monolithic catalysts offers multiple advantages in CO-PROX reactions, including a large geometric surface area that enhances exposure of active sites, short mass transfer pathways that improve apparent reaction rates, low pressure drops that reduce energy consumption, and high gas flow rates that promotes uniform distribution of gases over the catalyst surface [6,7]. Enhancing the performance of ceramic monolithic catalysts has been studied by optimizing monolith materials and channel structures, developing efficient active components and promoters, improving coating techniques, and utilizing advanced molding technologies [8,9,10]. Comprehensive studies have indicated that the design of monolithic catalysts should focus on adequately exposing active sites to improve catalytic efficiency, optimizing monolith structure and materials to enhance mass and heat transfer properties, and strengthening interactions between active sites and the monolith to improve thermal and mechanical stability under high temperature and high space velocity conditions [11,12,13]. Additionally, cost-effectiveness must be considered to promote feasibility in industrial applications. Therefore, advancing the materials and preparation methods of ceramic monolithic catalysts is essential to realize their practical application in CO-PROX reactions [14].

In the field of monolithic catalysts, Avila et al. [11] have summarized the preparation methods of monolithic catalyst substrates. Mitra et al. [12] have reviewed the coating methods of active components onto monolithic catalysts. Farrauto et al. [13] have provided a summary of the applications of monolithic catalysts in the field of hydrogen energy. Fu et al. [15,16] have reviewed the use of monolithic catalysts in the removal of volatile organic compounds (VOCs). However, no comprehensive review has summarized the study of monolithic catalysts in CO-PROX reactions, especially for ceramic monolithic catalysts. This work aims to systematically review advancements in the application of ceramic monolithic catalysts for CO-PROX reactions, including active sites such as precious metals and non-precious metals, monolith structure, and also coating strategies. Moreover, the structure–performance relationship and challenges faced in practical applications were also emphasized.

## 2. Active Site Regulation of Ceramic Monolithic Catalysts

### 2.1. Precious Metal-Based Ceramic Monolithic Catalysts

**Figure 1:**
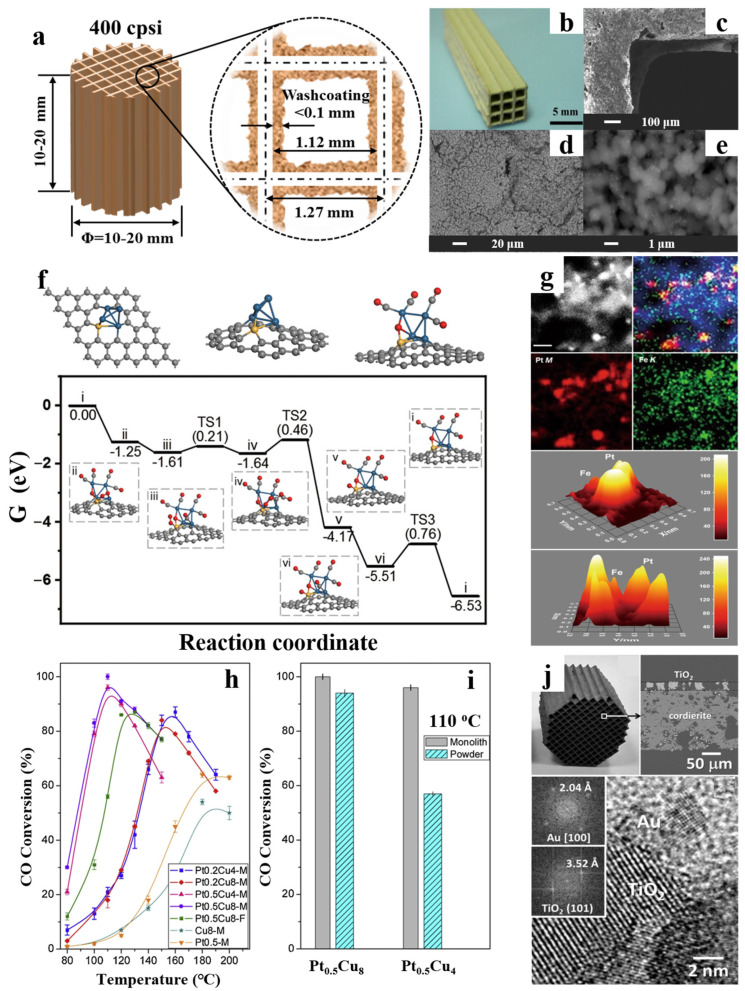
(**a**) Illustrations of the most commonly used ceramic monolithic catalysts; (**b**) photographs of the Pt/Z-PM based ceramic monolithic catalyst and (**c**–**e**) the corresponding SEM characterizations [18]; (**f**) DFT calculations of CO oxidation on Pt_0.75_Fe_0.2_/ND@G [19]; (**g**) EDX mapping images of the Pt_0.75_Fe_0.2_/ND@G catalyst [19]; CO-PROX reaction on PtCu/Al_2_O_3_ catalysts washcoated on a honeycomb monolith (M) and a foam monolith (F) [20]; (**h**) CO conversion; (**i**) comparative CO conversion bars of a PtCu/Al_2_O_3_ honeycomb monolithic catalyst and powder catalyst at 110 °C [20]; (**j**) SEM and HRTEM images of the Au/TiO_2_ ceramic monolithic catalyst [21].

Among CO-PROX ceramic monolithic catalysts, the coating of Pt-group metal catalysts on ceramic monoliths has always been a hot topic of research (Figure 1a–i). In a systematic screening of Pt-group metal ceramic monolithic catalysts, Zhou et al. [17] compared the performance of various Pt-group metal catalysts supported on γ-Al_2_O_3_, including Pt, Pd, Rh, and Ru. The study found that the Pt/γ-Al_2_O_3_ catalyst exhibited the best performance in the CO-PROX reaction, achieving a CO conversion of 95.7% at 170 °C with the outlet CO concentration below 20 ppm and showing no deactivation during a 1000 h stability test.

Following systematic screening of Pt-group metal catalysts, researchers have further explored the promotional effects of dopants on the performance of Pt-based catalysts. Korotkikh et al. [22] introduced non-precious metal oxide dopants into Selectoxo™ series Pt/γ-Al_2_O_3_ catalysts and coated them onto cordierite honeycomb ceramic monoliths. The promoted Pt catalyst achieved a CO conversion rate of 68%, while the catalyst without dopants only reached 13.2% at 90 °C with an O_2_/CO molar ratio of 0.5. The introduction of dopants provided active sites for the adsorption and dissociation of O_2_, effectively enhancing the activity and selectivity of the Pt catalyst in the CO-PROX reaction. Roberts et al. [23] further investigated the promotion effect of FeO_x_ dopants on Pt/γ-Al_2_O_3_ catalysts, which were coated onto cordierite honeycomb ceramics for performance testing. The CO conversion rate increased with the increase of Fe loading, and the catalyst containing 0.5 wt.% Fe achieved approximately 80% CO conversion and 40% selectivity at 100 °C with a feed of 1% CO and 1% O_2_. Many studies have revealed that the introduction of FeO_x_ can minimize the kinetic inhibition caused by CO adsorption on the Pt surface by providing active oxygen for CO oxidation (Figure 1f,g) [19].

To further enhance the activity of Pt-based catalysts, researchers have introduced other active metals to design multimetallic catalysts. Gómez et al. [20] prepared PtCu/Al_2_O_3_ catalysts and coated them on both cordierite and alumina foam, which exhibited high CO conversion in the temperature range of 110–130 °C, particularly with low Pt loading (0.2 and 0.5 wt.%) and high Cu content (4 and 8 wt.%) (Figure 1h,i). Notably, the Pt_0.5_Cu_8_ catalyst achieved a 100% CO conversion at 110 °C and demonstrated high tolerance to CO_2_ and H_2_O. The high CO conversions at low temperatures were likely due to the simultaneous formation of small Pt particles in close contact with Cu species. Zhang et al. [24] introduced both Cu and Fe into the Pt-based catalyst, which effectively reduced the CO content in industrial reformate gas to below 10 ppm under various inlet gas compositions, temperatures, and space velocity ratios within an inlet temperature range of 65–120 °C. The catalyst maintained high activity and selectivity even under high water vapor content (45%), demonstrating good water tolerance under actual reformate gas feed conditions at low temperatures (65–120 °C). These findings offer an effective CO removal strategy for low temperature PEMFCs.

The choice and optimization of the support play a crucial role in enhancing the performance and stability of ceramic monolithic catalysts. In previous studies, γ-Al_2_O_3_ was the most common support for Pt-based catalysts. Maeda et al. [25] achieved superior catalytic performance by replacing the traditional γ-Al_2_O_3_ with mordenite (MOR), which increased the selectivity of CO oxidation by suppressing hydrogen adsorption. By coating the Pt-Fe/MOR catalyst onto a cordierite honeycomb monolith, the CO concentration in the simulated reformate gas was reduced below 10 ppm under optimized conditions. Moreover, the monolithic catalyst exhibited high stability with no significant change in outlet CO concentration after 500 h of operation, which is highly significant for practical applications. However, after a certain period of CO-PROX reaction, the catalyst quickly deactivated, and the CO concentration increased due to condensation of H_2_O in the MOR pores at lower temperature ranges (100–120 °C). To address this issue, Maeda et al. [26] used hydrophobic silica sol instead of alumina sol during coating of Pt-Fe/MOR powder on the ceramic monolith. The monolithic catalyst using hydrophobic silica sol as the binder exhibited excellent water resistance and maintained a CO concentration of about 20 ppm after operating for 200 h under wet conditions, highlighting the importance of the binder on the catalytic performance of monolithic catalysts. In pursuit of economical catalytic materials, Neri et al. [18] developed a Pt catalyst using a new zeolite material (Z-PM) derived from mining waste pumice as the support (Figure 1b–e). This sustainably sourced material showed significant advantages in catalytic performance, achieving 100% O_2_ conversion at a low temperature of 100 °C, whereas the traditional Pt/SiO_2_ catalyst required nearly 200 °C.

Among the Pt-group metal catalysts, Ru-based catalysts have also shown potential in the industrial application of the CO-PROX reaction. Huang et al. [27] systemically explored the reaction mechanism of a Ru/Al_2_O_3_ ceramic monolithic catalyst by comparing it with a Pt/Al_2_O_3_ ceramic monolithic catalyst. The CO conversion of the Ru/Al_2_O_3_ ceramic monolithic catalyst was highly sensitive to the Ru content below 100 °C, with the 5% Ru/Al_2_O_3_ catalyst achieving three times the CO conversion of the 0.1% Ru/Al_2_O_3_ at 80 °C. In contrast, for Pt catalysts, the temperature at which maximum CO conversion was achieved decreased significantly with increased Pt loading. The 1% Pt/Al_2_O_3_ catalyst reached maximum CO conversion at 140 °C, while the 5% Pt/Al_2_O_3_ catalyst achieved this between 60–100 °C. Additionally, the experiments showed that the methane yield of Ru-based catalysts increased with both temperature and Ru content, while the methane yield of Pt-based catalysts was very low across the entire tested temperature range (60–240 °C).

According to the research of Huang et al. [27], adding Fe to the Ru/Al_2_O_3_ catalyst improved CO conversion in the temperature range of 60–160 °C, while adding Co enhanced CO conversion at temperatures above 200 °C and effectively suppressed methane yield. The CO conversion and methane yield were not sensitive to the pore density of the honeycomb monolith in most conditions under 100–140 °C. Ru-based catalysts can achieve the highest CO conversion and lower methane yields in specific temperature ranges with appropriate additives (such as Fe or Co), while Pt-based catalysts maintain a low methane yield over a wide temperature range. Therefore, the choice between these two types of metals should be based on specific industrial application requirements and operating conditions.

Research on CO-PROX reactions under low temperature conditions holds significant value for both fundamental scientific research and practical industrial applications. Among various catalysts, Au-based catalysts exhibit relatively high activity in low temperature CO oxidation reactions. Moreno et al. [21] investigated the performance of cordierite monolithic catalysts coated with Au/TiO_2_ (Figure 1j) in the CO-PROX reaction. A genetic algorithm was utilized to estimate the parameters of the nonlinear empirical model for this system. The predicted values from the model were fitted with the experimental values to verify its validity, providing a validated kinetic model for predicting the performance of the Au/TiO_2_ ceramic monolithic catalyst in the CO-PROX reaction under various operating conditions.

### 2.2. Non-Precious Metal-Based Ceramic Monolithic Catalysts

**Figure 2:**
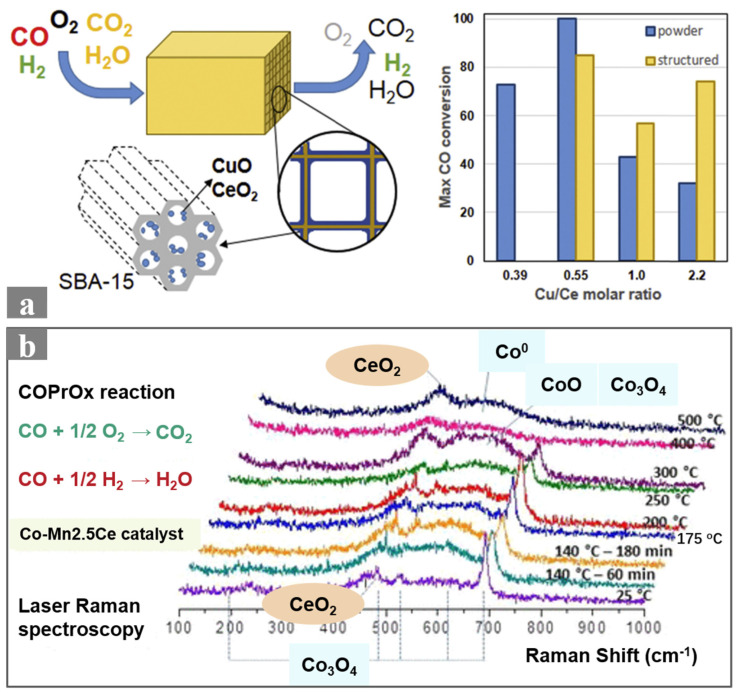
(**a**) Schematic diagram of CuO/CeO_2_ honeycomb ceramic monolithic catalyst and comparison of powder and ceramic monolithic catalyst activity with different Cu/Ce ratios [28]; (**b**) in situ Raman spectra of the MnCo/CeO_2_ catalyst at various temperatures during the CO-PROX reaction [32].

Traditional precious metal catalysts, though highly active, are costly and limited in resources. Therefore, developing non-precious metal catalysts, especially those with high activity, selectivity, and cost-effectiveness, is crucial for promoting their commercialization. CuO/CeO_2_ catalysts demonstrate unique performance in CO-PROX reactions due to the synergistic adsorption of CO and O_2_ on CuO and CeO_2_, respectively (Figure 2a). A series of studies have focused on optimizing the contents of the CuO and CeO_2_ active phases by coating CuO/CeO_2_ onto ceramic monolithic catalysts [28]. It was found that catalyst deposition on the monolithic walls improved the catalytic performance of CuCe-1.0 M and CuCe-2.2 M. According to Barbato et al. [29], the monolithic catalyst with a CuO/CeO_2_ molar ratio of 0.55 exhibited the best CO conversion, reaching 100% at 160 °C (Figure 2a). Ayastuy et al. [30] demonstrated that catalysts with 7% and 9% copper loading exhibited good activity and selectivity under conditions involving CO_2_ and H_2_O. Both studies indicated that the optimal operational temperature range for selective CO conversion was significantly influenced by CuO loading. Further research is needed to improve catalyst activity by gaining insight into the synergistic effect of CuO with CeO_2_ species in the CO-PROX reaction.

Boix et al. [31] investigated the effect of different types of SiO_2_ supports, including diatomaceous earth, commercial fumed silica, and synthesized mesoporous SBA-15, on CO-PROX performance. The results showed that the CuO/CeO_2_ catalyst supported on diatomaceous earth demonstrated excellent activity in the CO-PROX reaction, achieving over 90% CO conversion in the 140–210 °C temperature range and exceeding 99% at 160 °C. Moreover, the monolithic catalyst exhibited similar catalytic performance to its powder counterpart and maintained good chemical stability over the long term, even in the presence of CO_2_ and H_2_O. Characterizations revealed that the close contact and synergistic effects between CuO and CeO_2_ nanoparticles, as well as the formation of oxygen vacancies, are key factors in enhancing catalytic activity.

Cobalt oxides, due to their redox properties, have been recently studied for CO oxidation applications. Boix et al. [33] developed a Co/ZrO_2_ catalyst on cordierite honeycomb ceramic monoliths for CO-PROX, achieving 95% CO conversion and 60% selectivity at 230 °C, with activity unchanged over a 100 h stability test. Using a ZrO_2_ support forms an open flake-like structure on the monolithic catalyst coating, enhancing reactant diffusion to active sites. However, this structure leads to low mechanical stability of the coating. It is crucial to study how to improve catalytic activity while maintaining mechanical stability. Furthermore, Boix et al. [34] optimized catalyst performance by adding Mn dopants. Comparing the performance of MnCo/CeO_2_, Co/CeO_2_, and Co/ZrO_2_ catalysts coated on honeycomb ceramic monoliths, the MnCo/CeO_2_ ceramic monolithic catalyst exhibited the best CO conversion at low temperatures. The addition of Mn promoted re-oxidation of Co^2+^ to Co^3+^, thereby increasing CO oxidation abilities at low temperatures (Figure 2b) [32].

Until now, non-precious metal-based ceramic monolithic catalysts still perform inferior to their precious metal-based counterparts. Under the same conditions, non-precious metal catalysts typically show lower conversion compared to noble metal catalysts and require higher operating temperatures to reach comparable levels. Additionally, non-precious metal catalysts often face stability and durability challenges during prolonged use and can be adversely affected by CO_2_ and steam, leading to a decline in performance. Therefore, some novel operating strategies have been advanced to further enhance the performance of non-precious metal-based ceramic monolithic catalysts. For example, based on its abundant oxygen vacancy characteristics, Benedetto et al. [35] proposed a novel CO removal strategy using a CuO/CeO_2_ catalyst reactive trap. They found that the adsorption and desorption of CO could be effectively controlled by optimizing operational parameters, such as contact time and CO inlet concentration, to achieve efficient CO removal. Furthermore, Landi et al. [36] proposed a two-stage strategy for cordierite monolithic catalysts, leveraging the catalytic and adsorption properties of the CuO/CeO_2_ material. In this process, CO is first adsorbed and then converted into CO_2_ in the presence of O_2_. This approach not only achieved efficient CO removal but also avoided excessive use of O_2_, thus reducing H_2_ consumption.

## 3. Monolith Structure Regulation of Ceramic Monolithic Catalysts

The structure and properties of monoliths play a crucial role in determining the performance of ceramic monolithic catalysts, attracting significant attention in recent studies. Thin washcoating and small pore diameters can significantly reduce the internal diffusion limitations of ceramic monolithic catalysts. This optimization allows the gas to penetrate the entire catalyst pore network effectively, as thin layers and small pores provide a short diffusion path. Conversely, if the catalyst layer is too thick, it acts as a diffusion barrier, preventing efficient gas penetration into the deeper layers. Consequently, only the upper portion of the catalyst layer is utilized, leading to suboptimal catalyst efficiency [37,38,39,40]. Boix et al. [20] compared the mechanical stability of two types of ceramic monolith-supported PtCu catalysts and found that 77% of active mass remained adhered to the cordierite walls of the honeycomb monolith after 10 min of sonication, whereas only 20% of active mass remained attached to the foam monolith.

**Figure 3:**
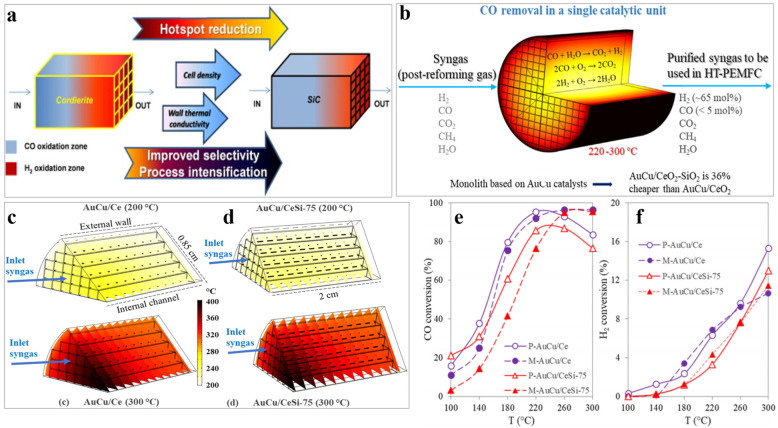
(**a**) Advantages of high cell density SiC as a replacement for honeycomb monoliths [42]; (**b**) schematic diagram of the AuCu monolithic catalyst; (**c**,**d**) temperature distribution across the cross-section of the monolithic catalyst under reaction temperatures of 200 °C and 300 °C, respectively [43]; conversion of (**e**) CO and (**f**) H_2_ obtained in CO-PROX over AuCu powder catalysts and monolithic catalysts [43].

The effect of monolith geometry and thermal conductivity on catalytic performance was systematically explored by Landi et al. [41]. Through a series of experimental and modeling studies [42], they found that the physical properties of the monolith, such as pore density and thermal conductivity, significantly impacted CO conversion and selectivity. Typically, SiC substrates with high pore density and higher thermal conductivity achieved better heat transfer than traditional cordierite honeycomb ceramic substrates in the CO-PROX process (Figure 3a).

Cobo et al. [43] investigated the impact of monolith length on CO-PROX performance using computational fluid dynamics (CFD) simulations. The study revealed a significant temperature gradient at the monolith inlet (Figure 3b,c), which then rapidly diminished along the channel. Increasing the monolith length can help stabilize the axial velocity of the airflow and convective heat transfer, thereby reducing the impact of the temperature gradient. Finally, the optimized AuCu/CeO_2_-SiO_2_ ceramic monolithic catalyst demonstrated superior activity at reaction temperatures above 260 °C compared to the powder catalyst (Figure 3d,e).

**Figure 4:**
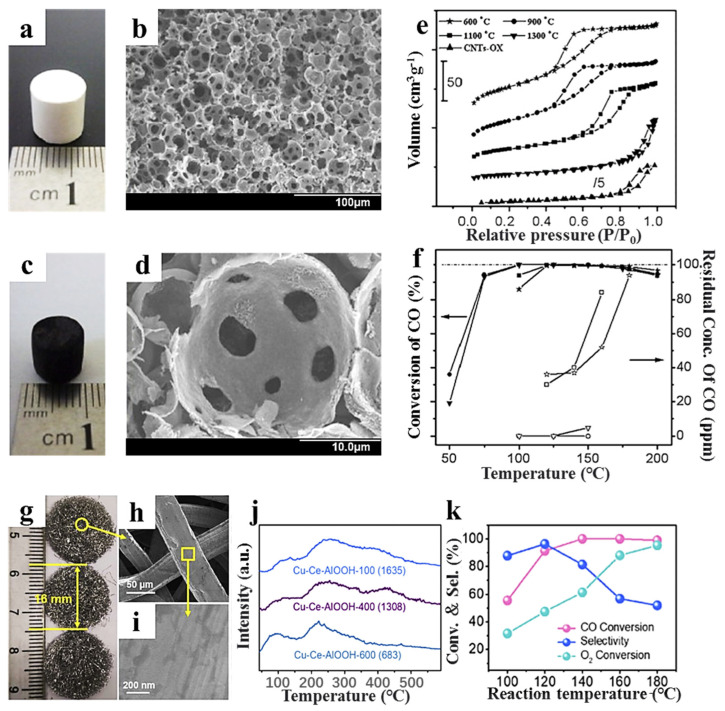
Photographs and SEM images of (**a**,**b**) macro-mesoporous hierarchical Al_2_O_3_ and (**c**,**d**) hierarchical CNT-Al_2_O_3_ [44]; (**e**) BJH pore size distribution curves of the hierarchical CNT-Al_2_O_3_ monolith calcined at different temperatures [44]; (**f**) reaction temperature dependence of CO conversion (filled symbol) and residual concentration of CO (open symbol) for the Pt-Ni/CNT-Al_2_O_3_ monolithic catalyst [44]; (**g**) photographs and (**h**,**i**) SEM image of pure Al-fiber [49]; (**j**) TPD of a CuO-CeO_2_/AlOOH/Al fiber monolithic catalyst calcined at different temperatures. The values in brackets are the areas of the second peak (180-580 °C) [49]; (**k**) CO-PROX catalytic performance of the CuO-CeO_2_/AlOOH/Al-100 fiber monolithic catalyst [49].

Some ceramic monoliths with unique structures have been designed to further improve performance. Liu et al. developed a macro-mesoporous hierarchical Al_2_O_3_ monolith using a templating method (Figure 4a,b). By coating different types of catalysts (Pt-Ni [44,45], CuO/CeO_2_ [46], Pt/γ-Al_2_O_3_, and K-Pt/γ-Al_2_O_3_ [47]), these catalysts exhibited higher CO conversion and O_2_ selectivity than powder catalysts, likely due to their macro-mesoporous hierarchical structure. Additionally, they incorporated carbon nanotubes into Al_2_O_3_ to create a novel composite material [44]. These composite materials possessed interconnected spherical macropores, adjusted mesopores, and a monolithic framework with carbon nanotubes uniformly distributed on the Al_2_O_3_ matrix (Figure 4c–e). As shown in Figure 4f, CO was purified completely at 100–150 °C in gases consisting of 1% CO with a volume space velocity of 10,400 h^−1^ for the Pt-Ni/CNT-Al_2_O_3_ monolithic catalyst. Berenguer-Murcia et al. [48] designed a capillary microreactor by incorporating hierarchical SiO_2_ monoliths into fused silica capillary tubes. These microreactors offer the advantages of flexibility, easy handling, and ease of scale-up. Yan et al. [49] developed a CuO-CeO_2_/AlOOH/Al fiber monolithic catalyst by growing AlOOH nanosheets on Al fibers through hydrothermal oxidation (Figure 4g–i). The AlOOH/Al fiber treated at 100 °C, with the highest hydroxyl content, exhibited optimal CuO-CeO_2_ dispersion and strong metal–support interaction, resulting in the highest CO conversion and O_2_ selectivity (Figure 4j,k).

## 4. Coating Strategies of Ceramic Monolithic Catalysts

The adhesion of coatings is another crucial factor significantly affecting the performance of ceramic monolithic catalysts. The washcoating method is a commonly used method for adhering catalyst powder to the monolith. Wu et al. [50] explored the impact of various factors during the washcoating process on the adhesion stability of catalyst coatings. They found that the adhesion of the coating could be significantly improved by optimizing preparation parameters such as the monolith substrate pretreatment agent, the properties of the coating solution, and the coating content, thereby enhancing the mechanical stability of the catalyst layer. The optimal preparation conditions included a 50 wt.% acetic acid pretreating agent, 25 wt.% solid content, 6 wt.% PEG-1000 additive, and two-time washcoating after calcination for high mechanical stability.

**Figure 5:**
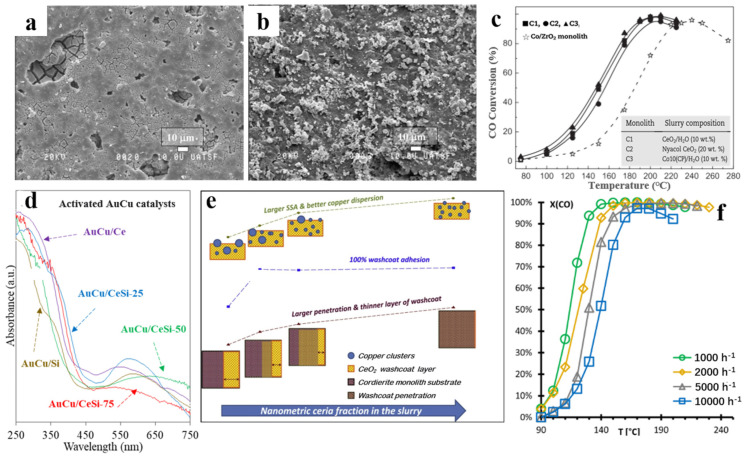
SEM images of monolithic catalysts prepared (**a**) using a slurry of co-precipitated Co/CeO_2_ (**b**) using CeO_2_ washcoating followed by Co impregnation [51]; (**c**) CO conversion obtained with the monolithic catalysts [51]; (**d**) UV-vis spectra for AuCu/CeO_2_ ceramic monolithic catalysts [43]; (**e**) influence of the modified dip coating method on coating adhesion [52]; (**f**) CO conversion at different space velocities for CuO/CeO_2_ ceramic monolithic catalysts via the urea–combustion method [53].

Boix et al. [51] investigated the effect of the washcoating sequence of active species on the performance and mechanical stability of ceramic monolithic catalysts. A sample prepared using a slurry of co-precipitated Co/CeO_2_ showed better performance than those obtained by CeO_2_ washcoating followed by Co impregnation, achieving 96% CO conversion and 60% selectivity for CO_2_ at 190 °C. The characterization results indicated that co-precipitation of metal oxides resulted in a smooth surface and better contact between oxide phases, thus enhancing redox capacity (Figure 5a,b). Additionally, catalysts with high Co loading (10 wt.% Co) demonstrated excellent CO conversion, with Co_3_O_4_ as the main active phase. In contrast, catalysts with low Co loading (below the solubility limit of Co in CeO_2_) showed poor performance, indicating that segregation of Co_3_O_4_ in the catalyst is beneficial (Figure 5c). Cobo et al. [43] explored the effect of SiO_2_ addition on the performance of AuCu/CeO_2_ ceramic monolithic catalysts. They discovered that incorporating SiO_2_ increased the surface area by up to 3.4 times and improved adhesion of the catalyst coating to the ceramic monolith walls (Figure 5d). The AuCu/CeO_2_-SiO_2_ ceramic monolith catalyst demonstrated superior activity at reaction temperatures above 260 °C compared to the powder catalyst. Barbato et al. [29] evaluated CO-PROX in a Cu/CeO_2_-based microreactor, suggesting that hotspots in the first part of the monolith channels (<1 mm) improve CO conversion and mitigate H_2_ combustion activation. Therefore, above 260 °C, the increased temperature in internal channels, promoted by the exothermic reaction and heat transfer in monolith channels, could be associated with improved CO oxidation activity in monolithic reactors compared to powder samples.

Besides the traditional washcoating method, researchers have also developed novel coating methods to improve the performance of ceramic monolithic catalysts. Landi et al. [52] developed a modified dip coating method to improve washcoat adhesion. Wet milling of the CeO_2_ powder used to prepare the slurry and the addition of colloidal CeO_2_ significantly improved washcoat adhesion due to partial penetration into the cordierite macropores (Figure 5e). Samples prepared with modified slurries also showed improved copper dispersion, resulting in higher selectivity to CO_2_. Meißner et al. [53] employed an in situ preparation method for CuO/CeO_2_ catalysts on ceramic monoliths via the urea–combustion method. The viscous precursor solution could penetrate the pores of the monolith, achieving deeper penetration and a better catalyst–substrate interaction than traditional methods. The calcination process further consolidated the catalyst coating structure, making it more uniform and robust. Therefore, the urea–nitrate combustion method not only increased catalyst loading but also enhanced adhesion on the monolith, improving overall performance and long-term stability. The as-prepared CuO/CeO_2_ monolithic catalysts can achieve CO conversion of more than 99%, in the temperature range of 180–220 °C at a GHSV of 1000 h^−1^ (Figure 5f).

## 5. Conclusions and Outlook

In summary, developing highly active and stable monolithic catalysts is crucial for industrial CO-PROX applications. The selection of active components, monolith structures, and preparation techniques significantly affects catalytic performance and durability (Table 1). This review highlights progress in applying ceramic monolithic catalysts for CO-PROX, focus on optimizing active species, the physical properties of monoliths, and coating methods. Pt-based ceramic monolithic catalysts are the most commonly used catalysts for the CO-PROX reaction, offering excellent activity and selectivity for CO oxidation. Among non-precious metal catalysts, CuO/CeO_2_ ceramic monolithic catalysts are particularly notable, achieving performance comparable to Pt-based catalysts, especially at high temperatures above 200 °C. The geometry, pore structure, length, and conductivity of monoliths play significant roles in determining CO-PROX performance by influencing heat and mass transfer properties. Designing monoliths with unique structures has great potential to further improve CO-PROX performance. Moreover, improved coating strategy beyond traditional washcoating are necessary to enhance the adhesion and properties of coatings, contributing to the stability of catalysts during long-term operation.

Future research on ceramic monolithic catalysts for CO-PROX reactions should focus on three main directions. Firstly, advancing monolith structures and coating methods to leverage the high heat and mass transfer efficiency of ceramic monoliths, including designing special channels, incorporating 3D printing technology, and improving coating corrosion resistance [54,55,56,57]. For example, Bueno-López et al. [55]. employed 3D printing to create honeycomb ceramic monoliths with asymmetric channels, enhancing gas turbulence and radial diffusion. Secondly, gaining deeper insights into the mechanisms that enhance CO-PROX ceramic monolithic catalyst performance. While many studies have focused on CO-PROX powder catalysts, the monolith process can cause structural and performance disparities. A thorough understanding of these differences can inform the rational construction of highly efficient monolithic catalysts [58,59]. Thirdly, promoting the commercialization of CO-PROX ceramic monolithic catalysts by selecting suitable monolith forms based on specific conditions and conducting long-term testing under realistic industrial conditions [60].

## Figures and Tables

**Table 1:** Comparative activity analysis of partial CO-PROX monolithic catalysts.

Catalyst	Carrier Type	Preparation Method	Reaction Conditions	T_50%_ (°C)	T_100%_ (°C)	#
Pt/Al_2_O_3_	Cordierite monoliths	Washcoating method	1% CO, 1% O_2_, 15% CO_2_, 20% H_2_O, 50% H_2_, N_2_ balance	180	180–230 (>90%)	[17]
5 wt.% Pt/0.5 wt.% Fe/γ-Al_2_O_3_	Cordierite monoliths	Washcoating method	42% H_2_, 9% CO_2_, 12% H_2_O, 1% CO, 1%O_2_, N_2_ balance	-	100 (80%)	[23]
Pt-Cu-Fe/Al_2_O_3_	Cordierite monoliths	Washcoating method	O_2_: 4500 ppm, 25% H_2_O, CO: 3000 ppm	-	80–100	[24]
Pt/Z-PM	Cordierite monoliths	Washcoating method	1% CO, 1.5% O_2_, H_2_ balance	75	/	[18]
1 wt.% Ru/γ-Al_2_O_3_	Cordierite monoliths	Washcoating method	0.5% CO, 2% O_2_, 28.5% CO_2_, 69% H_2_	140	150–200	[27]
Au/TiO_2_	Cordierite monoliths	Washcoating method	1.41% CO, 24.33% CO_2_, H_2_ balance, O_2_/CO = 0.4–4.1	100	/	[21]
CuO/CeO_2_	SiC monoliths	Washcoating method	CO/O_2_/H_2_ = 0.5/0.9/50	90	140–180	[41]
CuO/CeO_2_	SiC monoliths	Washcoating method	CO/O_2_/H_2_ = 0.5/0.9/50	90	120–140	[42]
AuCu/CeO_2_-SiO_2_	Cordierite monoliths	Washcoating method	19.9% H_2_, 6.3% CO, 5.2%CO_2_, 5.6% O_2_, 7.8% H_2_O, 55.2% N_2_	185	260–300	[43]
Pt-Ni	Macro-porous monolithic γ-Al_2_O_3_	Template method and washcoating method	1% CO, 1% O_2_, and 50% H_2_ in N_2_ balance	60	100–175	[45]
CuO/CeO_2_	Macro-porous monolithic γ-Al_2_O_3_	Template method and washcoating method	1% CO, 1% O_2_, 50% H_2_, 15% CO_2_, 8% H_2_O in N_2_	90	120–160	[46]
Pt/γ-Al_2_O_3_	Macro-porous monolithic γ-Al_2_O_3_	Template method and washcoating method	1% CO, 1% O_2_, 50% H_2_ in N_2_	180	225–275	[47]
K-Pt/γ-Al_2_O_3_	Macro-porous monolithic γ-Al_2_O_3_	Template method and washcoating method	1% CO, 1% O_2_, 50% H_2_ in N_2_	150	200–275	[47]
Pd/SiO_2_	Novel hierarchical SiO_2_ monolithic microreactors	Sol-gel method and impregnation method	2% CO, 2% O_22_, 30% H_2_, balance He.	160	/	[48]
Pt/SiO_2_	Novel hierarchical SiO_2_ monolithic microreactors	Sol-gel method and impregnation method	2% CO, 2% O_2_, 30% H, balance He.	190	/	[48]
CuO-CeO_2_	Al-fiber	Steam oxidation method and impregnation method	CO/O_2_/H_2_ = 0.5/0.5/49.5 (balance N_2_)	100	140–180	[49]
Co/CeO_2_	Cordierite monoliths	Washcoating method	1% CO, 1% O_2_, 40% H_2_, He balance	120	160	[51]
CuO/CeO_2_	Commercial honeycomb monoliths	Washcoating method	CO/O_2_/H_2_ = 0.5/0.9/50 (balance N_2_)	90	140–200	[52]
CuO/CeO_2_	Ceramic monolith	Direct coating	39% H_2_, 20% CO_2_, 1% CO, balance: N_2_	110	140–210	[53]
